# Acute Decompensated Heart Failure Is Routinely Treated as a Cardiopulmonary Syndrome 

**DOI:** 10.1371/journal.pone.0078222

**Published:** 2013-10-21

**Authors:** Kumar Dharmarajan, Kelly M. Strait, Tara Lagu, Peter K. Lindenauer, Mary E. Tinetti, Joanne Lynn, Shu-Xia Li, Harlan M. Krumholz

**Affiliations:** 1 Division of Cardiology, Columbia University Medical Center, New York, New York, United States of America; 2 Center for Outcomes Research and Evaluation, Yale-New Haven Hospital, New Haven, Connecticut, United States of America; 3 Center for Quality of Care Research, Baystate Medical Center, Springfield, Massachusetts, United States of America; 4 Division of General Internal Medicine and Geriatrics, Baystate Medical Center, Springfield, Massachusetts, United States of America; 5 Department of Medicine, Tufts University School of Medicine, Boston, Massachusetts, United States of America; 6 Program on Aging, Department of Medicine, Yale University School of Medicine, New Haven, Connecticut, United States of America; 7 Section of Chronic Disease Epidemiology, Yale School of Public Health, New Haven, Connecticut, United States of America; 8 Altarum Institute, Washington, District of Columbia, United States of America; 9 Section of Health Policy and Administration, Yale School of Public Health, New Haven, Connecticut, United States of America; 10 Robert Wood Johnson Clinical Scholars Program, Department of Medicine, Yale University School of Medicine, New Haven, Connecticut, United States of America; 11 Section of Cardiovascular Medicine, Department of Medicine, Yale University School of Medicine, New Haven, Connecticut, United States of America; Texas A & M, Division of Cardiology, United States of America

## Abstract

**Background:**

Heart failure as recognized and treated in typical practice may represent a complex condition that defies discrete categorizations. To illuminate this complexity, we examined treatment strategies for patients hospitalized and treated for decompensated heart failure. We focused on the receipt of medications appropriate for other acute conditions associated with shortness of breath including acute asthma, pneumonia, and exacerbated chronic obstructive pulmonary disease.

**Methods and Results:**

Using Premier Perspective^®^, we studied adults hospitalized with a principal discharge diagnosis of heart failure and evidence of acute heart failure treatment from 2009-2010 at 370 US hospitals. We determined treatment with acute respiratory therapies during the initial 2 days of hospitalization and daily during hospital days 3-5. We also calculated adjusted odds of in-hospital death, admission to the intensive care unit, and late intubation (intubation after hospital day 2). Among 164,494 heart failure hospitalizations, 53% received acute respiratory therapies during the first 2 hospital days: 37% received short-acting inhaled bronchodilators, 33% received antibiotics, and 10% received high-dose corticosteroids. Of these 87,319 hospitalizations, over 60% continued receiving respiratory therapies after hospital day 2. Respiratory treatment was more frequent among the 60,690 hospitalizations with chronic lung disease. Treatment with acute respiratory therapy during the first 2 hospital days was associated with higher adjusted odds of all adverse outcomes.

**Conclusions:**

Acute respiratory therapy is administered to more than half of patients hospitalized with and treated for decompensated heart failure. Heart failure is therefore regularly treated as a broader cardiopulmonary syndrome rather than as a singular cardiac condition.

## Introduction

Although heart failure is the most common cause of hospitalization for adults, it may in practice represent a more complex and less distinct entity than has been appreciated. While there has been growing recognition of multimorbidity [1] and the complexities associated with treating coexisting chronic conditions [2], scant attention has been paid to the degree to which our current constructs of decompensated heart failure fit with actual patterns of treatment. Accordingly, treatment guidelines [3,4] and clinical textbooks [5–8] regularly describe natural history and treatment from the perspective of a single disease or condition. Moreover, heart failure registries [9–11] and clinical investigations [12,13] have typically described the use of neurohormonal-blocking agents, inotropes, diuretics, and other cardiovascular agents without reporting on use of non-cardiac pharmacotherapies. Yet hospitalized patients in actual practice are complex, and their treatments may bridge traditional categories due to diagnostic uncertainty or coexisting acute illnesses [14]. 

In recent years, increasing attention has been dedicated to understanding the overlap of cardiac and pulmonary diseases. It has been shown that chronic lung and cardiac diseases are closely related, as chronic airflow obstruction can impair left ventricular filling, stroke volume, and cardiac output [15]. Moreover, the extent of pulmonary artery disease predicts both right ventricular function and exacerbations of chronic obstructive pulmonary disease [16]. These ties may carry over to the acute setting, where pulmonary conditions such as pneumonia can result in a spectrum of acute cardiac conditions such as decompensated heart failure, arrhythmia, and acute coronary syndromes [17].

Yet it is unknown how often patients hospitalized with decompensated heart failure receive concomitant treatment with acute respiratory therapies for conditions such as acute asthma, pneumonia, and exacerbated chronic obstructive pulmonary disease. Such co-treatment may be frequent in typical practice, as presenting symptoms and signs of heart failure are often non-specific [18,19] and are consistent with common acute and chronic respiratory conditions that are also frequently present [15,17,20,21]. Moreover, there is no singular pathognomonic finding or diagnostic test that differentiates heart failure from common respiratory conditions, which may frequently contribute to uncertainty in diagnosis [22]. 

We therefore hypothesized that patients hospitalized with decompensated heart failure would often receive concomitant treatment with acute respiratory therapies. To test this hypothesis, we investigated treatment strategies for patients hospitalized and treated for decompensated heart failure, focusing specifically on medications appropriate for other common conditions associated with symptoms of shortness of breath. Specifically, we sought to determine how often these patients are treated for acute asthma, pneumonia, and exacerbated chronic obstructive pulmonary disease, which may represent the ambiguity of diagnosis or the presence of more than 1 acute condition contributing to respiratory symptoms. We also determined the duration of the concomitant treatments and their association with adverse events. We characterized these outcomes among all heart failure hospitalizations meeting inclusion criteria as well as among the large [20] and higher risk [23,24] subset with chronic lung disease who we hypothesized would be even more likely to receive co-treatment for acute cardiac and pulmonary conditions. 

## Methods

### Ethics Statement

The Yale University Human Investigation Committee reviewed the study protocol and determined that it was not considered Human Subjects Research as defined by the Office of Human Research Protections. 

### Data Source and Study Sample

We conducted a retrospective cohort study using Perspective^®^, a voluntary, fee-supported database developed by Premier, Inc. for measuring quality and healthcare utilization. As of 2010, Perspective^®^ contained data on more than 130 million cumulative hospital discharges representing approximately 20% of annual acute care hospitalizations in the United States. In addition to information available in the standard hospital discharge file, Perspective^®^ contains a date-stamped log of all billed items at the patient level including diagnostic tests, medications, and therapeutic services. Premier has de-identified patient data in accordance with the United States Health Insurance Portability and Accountability Act and assigned a random identifier to each hospital. 

 We included hospitalizations from 2009 and 2010 for patients aged 18 years or older with a principal discharge diagnosis of heart failure as defined by International Classification of Diseases, Ninth Revision, Clinical Modification (ICD-9-CM) codes 402.01, 402.11, 402.91, 404.01, 404.03, 404.11, 404.13, 404.91, 404.93, and 428.xx). Although administrative codes are highly specific for decompensated heart failure [25–27], we sought to further increase the specificity of our study cohort by requiring hospitalizations to be at least 2 days in duration and include treatment with loop diuretics, intravenous vasodilators, or inotropes during hospital days 1 or 2. We excluded hospitalizations that involved transfers from another acute care facility or that had an unknown admission source, as information about treatment before hospitalization was unavailable. We further excluded hospitalizations with a pediatric attending to concentrate on care patterns of physicians who treat adults. 

### Treatments

For each hospitalization, we noted treatment with commonly used therapies for decompensated heart failure, acute asthma, pneumonia, and exacerbated chronic obstructive pulmonary disease. We chose to characterize treatments for these 3 respiratory conditions in particular, as they are among the most common causes of hospitalization due primarily to breathing difficulty [28]. For heart failure, we identified treatment with oral and intravenous loop diuretics, intravenous vasodilators including nitroglycerin, nitroprusside, and nesiritide, and inotropes including dobutamine, dopamine, and milrinone. For acute asthma, we noted use of short-acting inhaled bronchodilators including β-agonists, anti-cholinergics, and methylxanthine formulations. For pneumonia, we identified treatment with antibiotics from pharmacologic classes used to treat community-acquired and nosocomial pneumonia including penicillins, cephalosporins, fluoroquinolones, macrolides, vancomycin, tetracyclines, aminoglycosides, and carbapenems. For exacerbated chronic obstructive pulmonary disease, we identified daily corticosteroid treatments within a dose range of 20mg to 80mg of oral prednisone or 120mg to 800mg of intravenous prednisone equivalents, as has been done previously [29]. To increase the likelihood that antibiotics and corticosteroids were intended for respiratory illness, we used ICD-9-CM present-on-admission diagnosis codes to exclude hospitalizations with evidence of common infections besides pneumonia or inflammatory, allergic, or autoimmune conditions other than exacerbated chronic obstructive pulmonary disease (Tables S1 and S2 in File S1).

### Treatment Groups

**Figure 1:**
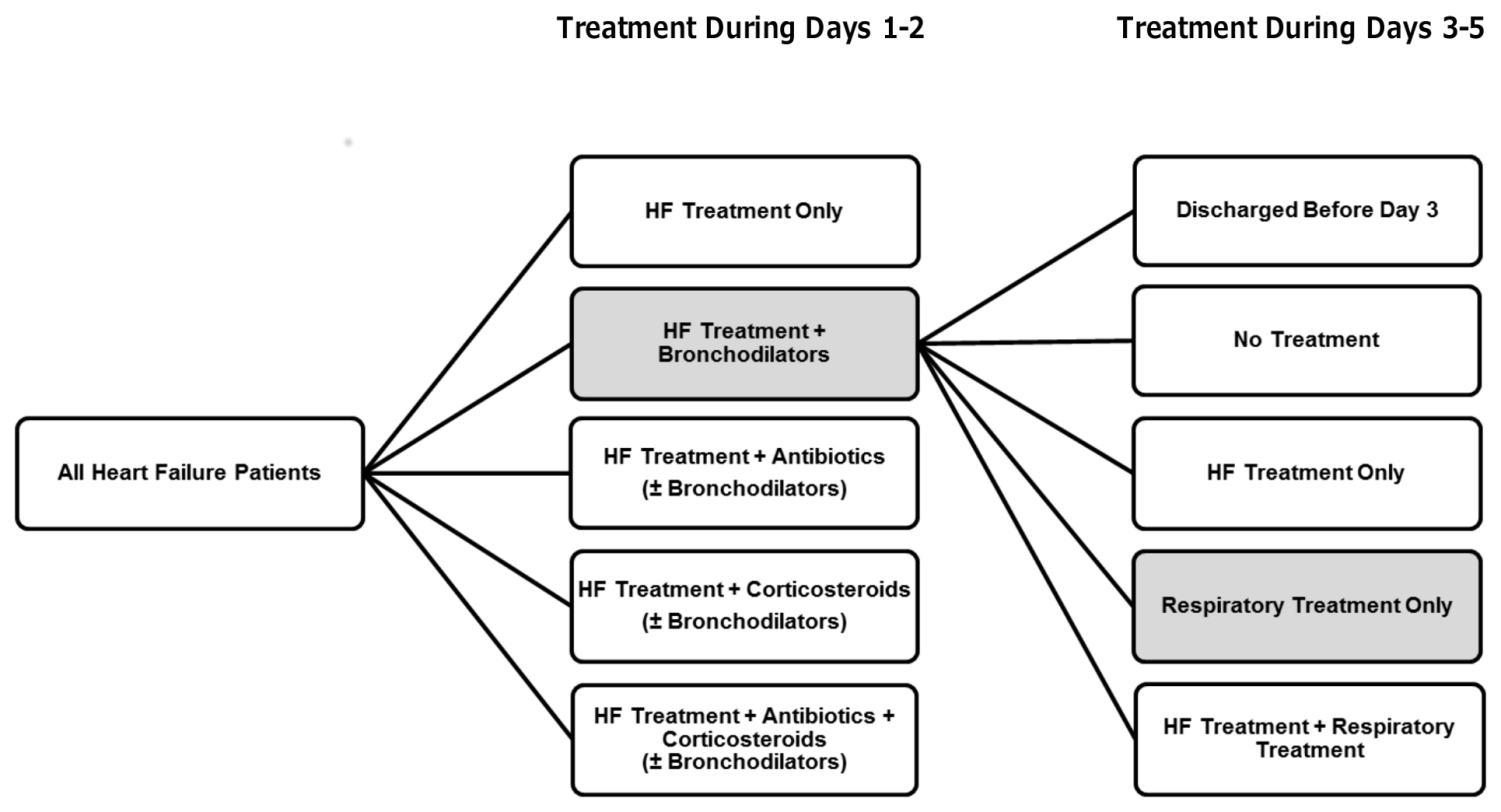
Longitudinal treatment pathways. A sample pathway is shown for a patient who receives treatment for heart failure and bronchodilators during the first 2 hospital days and acute respiratory treatment only during hospital days 3 through 5. Acute respiratory treatment includes treatment with short-acting inhaled bronchodilators, antibiotics, or corticosteroids. Within each time period, treatment groups are mutually exclusive and inclusive of all patients. HF, heart failure.

We assigned admissions to 1 of 5 treatment groups based on respiratory medications of interest that were dispensed during the first 2 days of hospitalization. Treatment groups were structured around potential treatment regimens for acute asthma, pneumonia or exacerbated chronic obstructive pulmonary disease as described by clinical guidelines or found in common use [30–33]. Treatment groups were also mutually exclusive and exhaustive, meaning that all hospitalizations meeting study inclusion criteria were able to be categorized into 1 of these 5 groups. These 5 initial treatment groups were defined by the receipt of (1) heart failure treatment only; (2) heart failure treatment plus inhaled bronchodilators only; (3) heart failure treatment plus antibiotics with or without inhaled bronchodilators; (4) heart failure treatment plus corticosteroids with or without inhaled bronchodilators; and (5) heart failure treatment plus antibiotics and corticosteroids with or without inhaled bronchodilators (Figure 1). 

To better understand the use of respiratory treatments over the period of hospitalization, we assigned each patient to a continuing treatment group if treatments were received each day during hospital days 3 through 5 or on the day of discharge, whichever occurred first. These 5 continuing treatment categories, which were also mutually exclusive and exhaustive, were (1) discharge after hospital day 2 (ineligible for continuing therapy); (2) no daily heart failure or respiratory treatments during days 3 through 5; (3) heart failure treatment only; (4) acute respiratory treatment only; and (5) heart failure treatment plus acute respiratory treatment (Figure 1). We assigned patients to longitudinal treatment pathways based on their combination of treatment during hospital days 1 to 2 and 3 through 5. 

For interested readers, further subdivision of continuing treatment categories for (1) acute respiratory treatment only and (2) heart failure treatment plus acute respiratory treatment is described in Figure S1 in File S1. 

### Outcomes

Our primary outcome was the percentage of heart failure hospitalizations that received common heart failure pharmacotherapies plus other respiratory therapies during the initial 2 hospital days. The consistency of findings was tested in subgroup analyses. We also noted heart failure and respiratory treatments dispensed daily during hospital days 3 through 5 for patients in each of the 5 initial treatment groups to characterize the relative frequency of different longitudinal treatment pathways. 

Secondary outcomes for each initial treatment group included in-hospital death, admission to an intensive care unit, and late intubation (intubation 2 or more days after admission). 

All analyses were repeated for the subgroup of patients with chronic lung disease as defined by Elixhauser and colleagues [34] to predominantly include chronic asthma and chronic obstructive pulmonary disease.

### Statistical Analyses

We calculated summary statistics for categorical variables using frequencies and percentages. Hierarchical logistic regression was used to calculate adjusted odds ratios for all outcomes. We used all hospitalizations when calculating odds ratios for admission to an intensive care unit and late intubation. We used 1 random hospitalization per patient when calculating odds ratios for in-hospital mortality, as death could only occur once. We adjusted for patient characteristics and Elixhauser comorbidities, with the exception of heart failure and chronic lung disease. Comorbidities were identified with software provided by the Healthcare Cost and Utilization Project of the Agency for Healthcare Research and Quality (versions 3.4, 3.5, and 3.6 for federal fiscal years 2009, 2010 and 2011, respectively) [34]. We calculated odds ratios and 95% confidence intervals for each outcome using as the referent the group initially receiving heart failure treatment only. Analyses were conducted with SAS 9.2 (SAS Institute Inc., Cary, NC). 

## Results

We identified 164,494 qualifying hospitalizations for heart failure among 370 hospitals. We did not include 26,382 hospitalizations with a principal discharge diagnosis of heart failure that failed to meet minimum length of stay requirements and a further 18,319 hospitalizations that did not meet treatment requirements. Among the 164,494 qualifying hospitalizations, we identified 127,410 unique patients, of whom 103,534 (81%) were hospitalized once, 16,442 (13%) were hospitalized twice, and 7,434 (6%) were hospitalized 3 or more times*.*

Of 164,494 qualifying heart failure hospitalizations, 63,690 (39%) had chronic lung disease (Table 1). Among the 63,690 qualifying heart failure hospitalizations with chronic lung disease, we identified 50,982 unique patients, of whom 42,685 (84%) were hospitalized once, 5,765 (11%) were hospitalized twice, and 2,532 (5%) were hospitalized 3 or more times. Patients with chronic lung disease were very similar in age, sex, and comorbidity profile to the overall heart failure cohort, though were more often obese (Table 1). 

**Table 1:** Patient Characteristics.

**Characteristic**		**All Heart Failure Hospitalizations (N=164,494**)	**Heart Failure Hospitalizations with Chronic Lung Disease (N=63,690)**
		**N (%)**	**N (%)**
**Age**	**18-54**	19,596 (12)	6,266 (10)
	**55-64**	23,670 (14)	9,927 (16)
	**65-74**	33,757 (21)	15,368 (24)
	**75-84**	47,392 (29)	19,438 (31)
	**85+**	40,079 (24)	12,691 (20)
**Sex**	**Female**	81,298 (49)	31,317 (49)
	**Male**	83,196 (51)	32,373 (51)
**Elixhauser Comorbidities**	**Peripheral Vascular Disease**	22,344 (14)	10,218 (16)
	**Hypertension**	115,379 (70)	44,652 (70)
	**Diabetes with and without Complications**	76,313 (46)	30,380 (48)
	**Obesity**	28,506 (17)	13,210 (21)
	**Chronic Pulmonary Disease**	63,690 (39)	63,690 (100)
	**Renal Failure**	69,781 (42)	26,843 (42)
	**Fluid and Electrolyte Disorders**	49,680 (30)	20,158 (32)
	**Deficiency Anemias**	54,237 (33)	21,824 (34)
	**Liver Disease**	4,112 (3)	1,612 (3)
	**Depression**	16,639 (10)	7,607 (12)

Acute respiratory therapy was common throughout hospitalization. Among our primary sample of all heart failure hospitalizations, 53% and 49% received treatment with at least 1 acute respiratory therapy during the first 2 hospital days and hospital days 3 through 5, respectively. Among heart failure hospitalizations with chronic lung disease, 73% and 67% received treatment with at least 1 acute respiratory therapy during the first 2 hospital days and hospital days 3 through 5, respectively.

**Figure 2:**
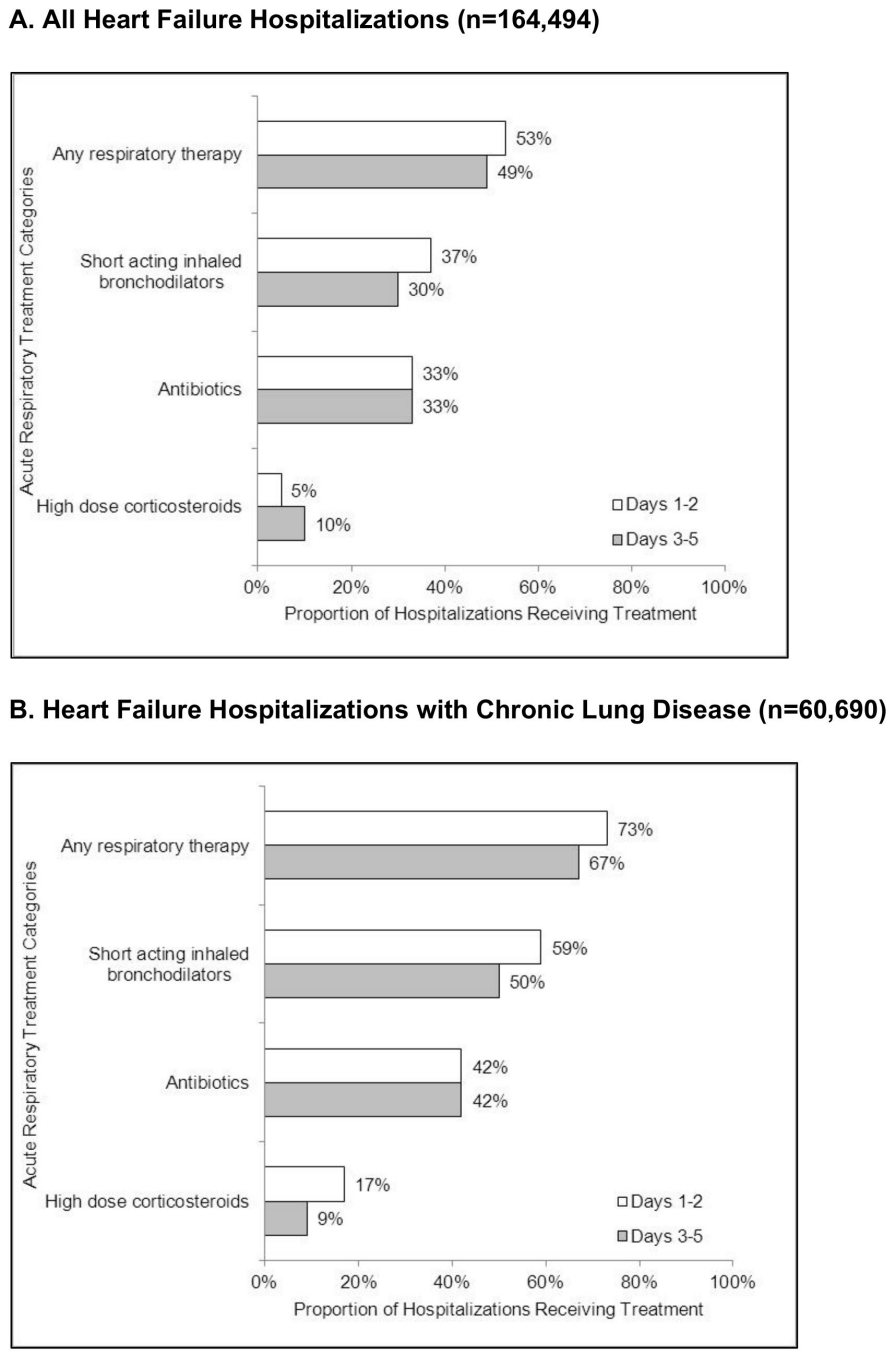
Proportion of hospitalizations receiving short-acting inhaled bronchodilators, antibiotics, and high-dose corticosteroids during the first 2 hospital days and hospital days 3 through 5. Figure A shows results for all heart failure hospitalizations (n=164,494). Figure B shows results for heart failure hospitalizations with chronic lung disease (n=60,690). HF, heart failure.

Treatment with short-acting inhaled bronchodilators and antibiotics was particularly frequent. Among the cohort of all heart failure hospitalizations, 37% and 30% received treatment with short-acting inhaled bronchodilators during the first 2 hospital days and hospital days 3 through 5, respectively (Figure 2A). Among the subset with chronic lung disease, 59% and 50% received treatment with short-acting inhaled bronchodilators during the first 2 hospital days and hospital days 3 through 5, respectively (Figure 2B). Inhaled beta agonists were the most frequently used agents in both cohorts during both time periods (Table S3 in File S1). Analogously, among the cohort of all heart failure hospitalizations, approximately one-third received antibiotics during both time periods (Figure 2A). Among heart failure hospitalizations with chronic lung disease, 42% received antibiotics during both time periods (Figure 2B). The most commonly used antibiotic pharmacologic classes were fluoroquinolones and cephalosporins in both cohorts during both time periods (Table S3 in File S1). Although corticosteroids were the least frequently used of all studied acute respiratory therapies, we did find that 17% of hospitalizations with chronic lung disease received high-dose corticosteroids during the first 2 hospital days (Table S3 in File S1). 

Further subgroup analyses demonstrated the consistency of findings across diverse populations (Table S4 in File S1). We found that at least 47% of patients received respiratory treatment during the first 2 days of hospitalization regardless of age. The rate of initial co-treatment remained 41% even in those without chronic lung disease. More than 50% of patients received initial respiratory treatment regardless of whether daily loop diuretics were given during only the first 2 hospital days or through day 5 of hospitalization. Analogously, rates of initial co-treatment were above 50% whether or not early diagnostic testing with natriuretic peptides was performed and above 43% whether or not early diagnostic testing with chest radiographs was performed. 

**Figure 3:**
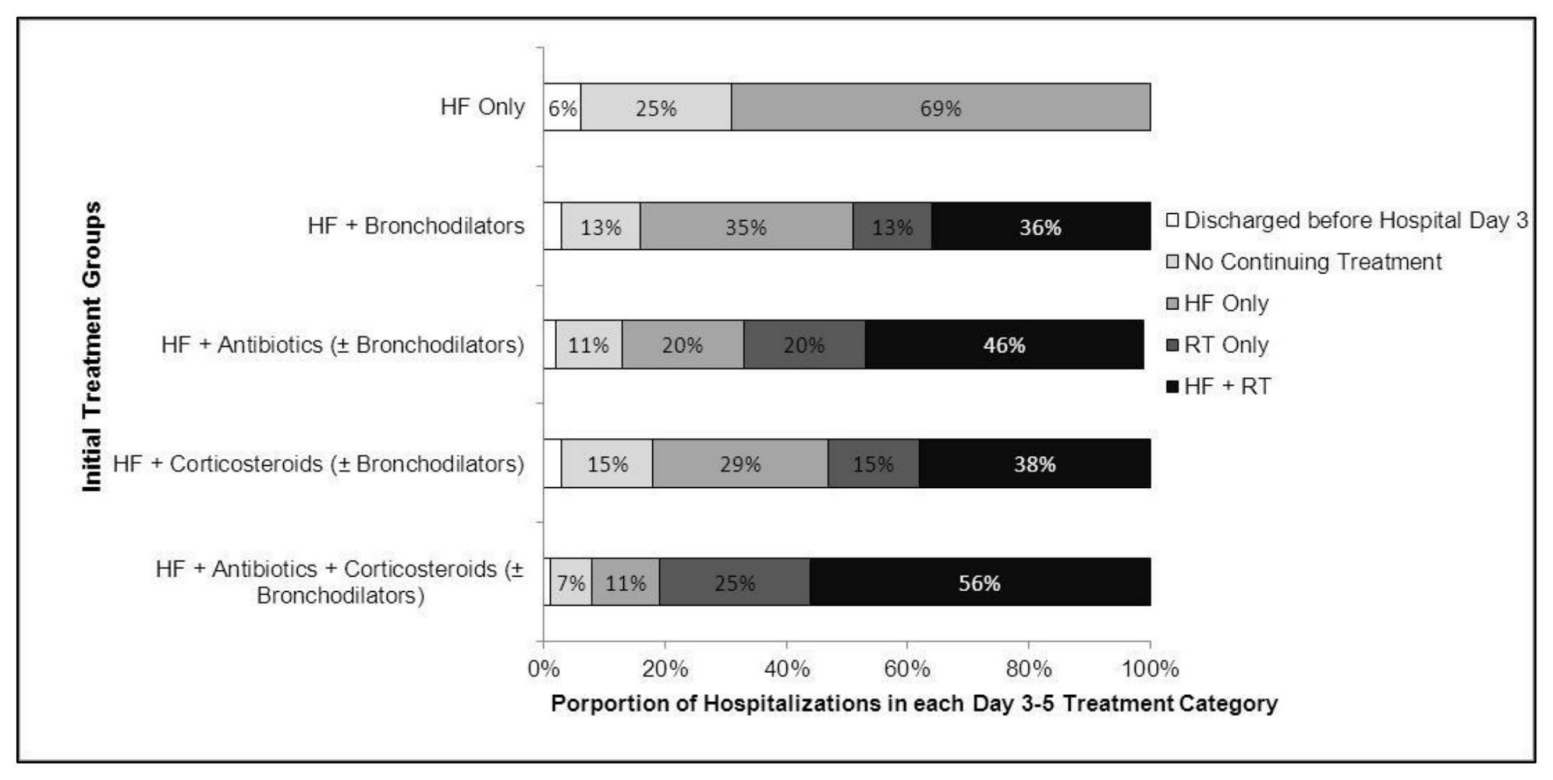
Daily treatment during hospital days 3 through 5 for patients in each initial treatment group. Percentages reflect the division of patients within each of the 5 initial treatment groups into 1 of 5 continuing treatment pathways. Pathways are mutually exclusive and exhaustive of all patients within the study sample. Non-labeled categories have percentages less than 5%. The 5 initial treatment groups were defined by the receipt during the first 2 hospital days of (1) heart failure treatment only (HF only); (2) heart failure treatment plus inhaled bronchodilators only (HF + Bronchodilators); (3) heart failure treatment plus antibiotics with or without inhaled bronchodilators (HF + Antibiotics (± Bronchodilators)); (4) heart failure treatment plus corticosteroids with or without inhaled bronchodilators (HF + Corticosteroids (± Bronchodilators)); or (5) heart failure treatment plus antibiotics and corticosteroids with or without inhaled bronchodilators (HF + Antibiotics + Corticosteroids (± Bronchodilators)). Treatment during hospital days 3 through 5 could fall into 1 of the 5 following categories: (1) discharge after hospital day 2 so ineligible for continuing therapy; (2) no daily heart failure or respiratory treatments during days 3 through 5; (3) daily heart failure treatment only (HF only); (4) daily acute respiratory treatment only (RT only); and (5) daily heart failure treatment plus daily acute respiratory treatment (HF + RT). HF, heart failure; RT, respiratory therapy.

Figure 3 describes the diversity of longitudinal treatment strategies for the entire sample of heart failure hospitalizations (n=164,494). Among the 77,175 hospitalizations that received initial treatment only for heart failure, less than 1% initiated daily respiratory treatment after hospital day 2. Among the 27,980 hospitalizations with initial heart failure and bronchodilator treatment, at least 1 respiratory treatment was continued daily after hospital day 2 in almost 50% of cases. Analogously, among the 44,846 hospitalizations receiving initial heart failure and antibiotic treatment, daily respiratory therapy was continued after hospital day 2 in 66% of cases. Finally, among the 8,641 hospitalizations receiving initial treatment with both antibiotics and corticosteroids, 81% continued to receive daily respiratory treatment after hospital day 2. Overall, among the 87,319 hospitalizations initially receiving respiratory treatment during the first 2 hospital days, more than 60% (n=53,615) continued to receive at least 1 respiratory treatment on a daily basis after day 2. For interested readers, greater detail about specific respiratory treatments received after hospital day 2 is presented in Table S5 in File S1.

Notably, among hospitalizations receiving at least 1 initial respiratory treatment, 18% continued to receive daily respiratory therapies in the absence of daily heart failure therapy after hospital day 2. For example, 19% of hospitalizations that initially received antibiotics with or without bronchodilators continued receiving daily respiratory therapy in the absence of daily treatment for heart failure after hospital day 2 (Table S5 in File S1). 

Observed rates of adverse outcomes among all heart failure hospitalizations were 2% for in-hospital death, 17% for admission to an intensive care unit, and 3% for late intubation. The median length of stay was 4 days (interquartile range 3, 7). Observed rates of adverse outcomes among heart failure hospitalizations with chronic lung disease were 3% for in-hospital death, 18% for admission to an intensive care unit, and 4% for late intubation. The median length of stay was 5 days (interquartile range 3, 7).

Adjusted odds for adverse outcomes consistently demonstrated an association between hospitalizations involving initial respiratory treatment and higher in-hospital mortality, admission to an intensive care unit, and late intubation compared with hospitalizations receiving only heart failure treatment. For example, among the cohort of all heart failure hospitalizations (Table 2), adjusted odds ratios with corresponding 95% confidence intervals for inpatient death were 1.74 (1.60, 1.88) and 2.04 (1.78, 2.34) for hospitalizations involving heart failure treatment plus antibiotics and heart failure treatment plus antibiotics and corticosteroids, respectively. Analogously, among the subset of heart failure hospitalizations with chronic lung disease (Table 3), adjusted odds ratios for inpatient death were 1.34 (1.17, 1.54) and 1.63 (1.36, 1.95) for hospitalizations involving heart failure treatment plus antibiotics and heart failure treatment plus antibiotics and corticosteroids, respectively. We similarly found that the adjusted odds of admission to an intensive care unit and late intubation were higher among patients receiving initial respiratory treatment in both the entire cohort of heart failure hospitalizations and the subgroup with chronic lung disease (Tables 2 and 3, respectively). 

**Table 2:** Odds Ratios for Adjusted Outcomes by Treatment During the First 2 Hospital Days for All Heart Failure Hospitalizations.

**Treatment During First 2 Hospital Days**	**Adjusted Outcomes by Treatment Group for All Heart Failure Hospitalizations (N=164,494)**		
	**Odds Ratio (95% CI)**		
	**In-Hospital Mortality**	**ICU admission**	**Late Intubation**
HF Only	--	--	--
HF + Bronchodilators	1.56 (1.41, 1.73)	1.27 (1.22, 1.33)	1.35 (1.24, 1.46)
HF + Antibiotics (± Bronchodilators)	1.74 (1.60, 1.88)	1.74 (1.68, 1.80)	1.76 (1.65, 1.88)
HF + Corticosteroids (± Bronchodilators)	1.40 (1.16, 1.71)	1.44 (1.33, 1.55)	1.33 (1.15, 1.54)
HF + Antibiotics + Corticosteroids (± Bronchodilators)	2.04 (1.78, 2.34)	2.03 (1.91, 2.16)	1.85 (1.65, 2.07)

HF: heart failure; ICU: intensive care unit.

**Table 3:** Odds Ratios for Adjusted Outcomes by Treatment During the First 2 Hospital Days for Heart Failure Hospitalizations with Chronic Lung Disease.

**Treatment During First 2 Hospital Days**	**Adjusted Outcomes by Treatment Group for Heart Failure Hospitalizations with Chronic Lung Disease (N=63,690)**		
	**Odds Ratio (95% CI)**		
	In-Hospital Mortality*	**ICU admission**	**Late Intubation**
HF Only	--	--	--
HF + Bronchodilators	1.24 (1.06, 1.45)	1.14 (1.07, 1.22)	1.11 (0.99, 1.25)
HF + Antibiotics (± Bronchodilators)	1.34 (1.17, 1.54)	1.71 (1.61, 1.81)	1.51 (1.36, 1.67)
HF + Corticosteroids (± Bronchodilators)	1.33 (1.04, 1.68)	1.32 (1.20, 1.46)	1.16 (0.97, 1.38)
HF + Antibiotics + Corticosteroids (± Bronchodilators)	1.63 (1.36, 1.95)	1.87 (1.73, 2.02)	1.60 (1.40, 1.83)

HF: heart failure; ICU: intensive care unit.

* Variables selected for in-hospital mortality model using step-wise selection.

## Discussion

Our study characterizes the substantial extent to which patients hospitalized with and treated for decompensated heart failure also receive therapy for acute respiratory conditions. Most patients receive acute respiratory therapies including short-acting inhaled bronchodilators, antibiotics, and corticosteroids during the first 2 hospital days. Slightly less than half of patients are treated exclusively for heart failure. When given early during hospitalization, respiratory therapies are continued after day 2 more than 60% of the time. These significant rates of co-treatment for acute cardiac and respiratory conditions were even higher among the large subset of patients with chronic lung disease. Over 70% of these patients received at least 1 acute respiratory therapy during hospitalization. These findings demonstrate that in typical practice within the United States, in contradistinction to contemporary teaching, heart failure is regularly treated as a broader cardiopulmonary syndrome rather than as a singular cardiac condition. The reasons underlying these treatment patterns and their appropriateness will require further explication. 

Co-treatment for acute cardiac and pulmonary disease appears to be a widespread phenomenon across diverse patient subgroups hospitalized with decompensated heart failure. For example, regardless of age and likelihood of having left ventricular systolic dysfunction [35], approximately 50% of hospitalizations involved treatment with acute respiratory therapy. More than 40% of hospitalizations involved respiratory therapy even in the absence of comorbid chronic lung disease. Perhaps most importantly, co-treatment rates were not different between patients treated with loop diuretics throughout hospitalization and those who did not receive these agents on a daily basis after hospital day 2, implying that the extent of volume overload was not clearly associated with the decision to broaden treatment to include respiratory therapies. 

Our findings suggest several possible explanations for the high frequency of co-treatment, all of which illustrate the complexity of heart failure in typical practice. First, it is possible that clinical presentation was often non-specific, as acute respiratory therapy was stopped after hospital day 2 in more than 20% of hospitalizations, presumably because an underlying diagnosis of heart failure became more apparent. This difficulty in differentiating heart failure from other conditions that cause respiratory symptoms is further evidenced by the fact that rates of co-treatment were above 50% even among patients who underwent early diagnostic testing with natriuretic peptides or chest radiographs. Second, many patients may have had evidence of true coexisting acute cardiac and pulmonary conditions, as respiratory treatment was regularly continued beyond the first 2 hospital days and was also associated with higher adverse outcomes including mortality. For these patients, the signs and symptoms of acute illness may have defied easy description, and there may have been evidence of more than 1 condition with an uncertain pathophysiologic basis. Yet our findings cannot determine the reasons for co-treatment or the appropriateness of this treatment strategy. Further study will be needed to elucidate the motivations for administering acute respiratory treatment to patients with suspected heart failure and the influence of comorbid lung disease on treatment choices.

Additional study is also needed to understand the benefits and harms of acute respiratory treatment in the heart failure population including potential differences in treatment effects for persons with chronic pulmonary disease. For example, it is unknown whether bronchodilators improve symptoms in persons with dyspnea stemming exclusively from acute heart failure as they may in patients with chronic heart failure [36,37]. It is also uncertain as to whether beta agonists in particular may have positive hemodynamic effects in the acute setting as has been shown in chronic disease [36,38]. These salutatory effects may be counter-balanced by an increased likelihood of arrhythmias [39], ischemia [40], or possible long term adverse consequences from sympathetic stimulation [41]. The effects of beta agonist therapy may also relate to the concomitant use of cardioselective as compared with non-cardioselective beta blockers [42]. Analogously, corticosteroid administration may result in enhanced diuresis among patients who are diuretic resistant [43]. However, glucocorticoids may also cause arrhythmias [44] and ischemic events [45]. Antibiotics can result in harmful drug interactions with commonly used cardiac medications [46] and severe nosocomial infections [47]. Potential harms may explain our finding consistent association of acute respiratory treatment with adverse outcomes. It may also be that patients receiving acute respiratory treatment are inherently more complex with greater severity of illness. These associations are therefore primarily hypothesis generating and merit prospective testing given the sizable numbers of heart failure patients co-treated for acute cardiac and pulmonary disease. At this time, acute respiratory treatment can at best be considered a marker of risk among heart failure hospitalizations.

Do the treatment patterns represent miscoding of heart failure? This possibility seems unlikely, as all patients were required to have treatments for heart failure, findings were consistent across subgroups defined by patient characteristics, diagnostic testing strategies, and interventions, and the number of patients broadly treated was far too large to plausibly result primarily from inaccurate coding. Moreover, studies have found ICD-9-CM codes for heart failure to be more than 95% specific with high positive predictive value relative to chart review or clinical assessment [25–27]. These codes have been used to identify epidemiologic trends in heart failure hospitalization [48] and evaluate hospital performance for the purposes of public reporting [49–51]. Moreover, ICD-9-CM codes for heart failure may be more specific than commonly used diagnostic criteria for decompensated heart failure including the Framingham, modified Boston, and Gothenburg indices [52]. Finally, our resultant cohort was similar to the patient population in a large dedicated heart failure registry with regard to patient comorbidities, use of intravenous loop diuretics, admission to the intensive care unit, and average length of stay [9–11]. 

A number of issues merit consideration when interpreting our results. First, our administrative database lacked information on vital signs, results of laboratory testing, left ventricular ejection fraction, and medication use both before and after hospitalization, thereby limiting our ability to define the reasons for respiratory treatment, its association with cardiac structural dysfunction, and the relationship of acute respiratory therapies with baseline treatment strategies. However, our objective was to define inpatient patterns of treatment, and this database is ideal for that purpose. Second, as we assessed a broad list of antibiotics that could be used for the treatment of pneumonia, it is possible that they were used for a different purpose. Nevertheless, we may have actually underestimated overall antibiotic and corticosteroid use in the inpatient treatment of patients with heart failure, as we excluded hospitalizations that had evidence of common infections besides pneumonia and inflammatory, allergic, or autoimmune conditions other than exacerbated chronic obstructive pulmonary disease. Third, short-acting inhaled bronchodilators may have been used for patient comfort or wheezing (cardiac asthma) in the setting of dyspnea rather than for treatment of another entity. However, usage of inhaled bronchodilators for this indication would not be consistent with heart failure guidelines [3,4]. Fourth, despite their wide geographic distribution and diverse range of structural characteristics, it is possible that hospitals in the Premier network are not generalizable to all US hospitals. Fifth, it may be that heart failure hospitalizations in the Perspective^®^ database are not adequately representative of all US heart failure hospitalizations. However, we found that patient characteristics, treatments received, and in-hospital outcomes among our study cohort are similar to that of hospitalizations within a large and well-respected US heart failure registry [9–11]. Finally, it remains possible that our estimates of acute respiratory treatment were biased upward by the inclusion of patients with primary respiratory conditions rather than decompensated heart failure. However, our estimates of co-treatment are sufficiently high to make it implausible that acute respiratory treatment is simply a byproduct of misclassification. Even if our findings were adjusted downward by a factor of 2, we would still find that over one-quarter of all heart failure hospitalizations and one-third of heart failure hospitalizations with chronic lung disease receive acute respiratory treatment during hospitalization.

We have demonstrated for the first time in a large study across many institutions that treatment with acute respiratory therapies occurs in more than one-half of patients hospitalized with decompensated heart failure. When started early during hospitalization, these respiratory treatments are continued after day 2 in the majority of cases. Rates of acute respiratory treatment are even higher among the large subgroup of patients with a history of chronic lung disease. These findings demonstrate that patients with heart failure are regularly treated with a broader cardiopulmonary treatment strategy throughout hospitalization. Further prospective investigation will be needed to better understand the motivations for this common approach to treatment and the extent to which it impacts patient outcomes. 

## Supporting Information

File S1 **Contains Tables S1-S5 and Figure S1.** Table S1. Infections Other Than Pneumonia Potentially Requiring Antibiotic Treatment.
Table S2. Conditions Other Than Exacerbated Chronic Obstructive Pulmonary Disease Potentially Requiring High-Dose Steroid Treatment.
Figure S1. Longitudinal Treatment Pathways. A sample pathway is shown for a patient who receives treatment for heart failure and bronchodilators during the first 2 hospital days and bronchodilators only during hospital days 3 through 5. Within each time period, treatment groups are mutually exclusive and inclusive of all patients. HF, heart failure.
Table S3. Treatments Received During the First 2 Hospital Days and Hospital Days 3 through 5.
Table S4. Acute Respiratory Treatment During the First Two Days of Hospitalization by Patient Subgroup.
Table S5. Longitudinal Treatment Pathways: Treatment During Hospital Days 3 through 5 for Patients within Each Initial Treatment Group.
(DOCX)

## References

[1] Barnett K, Mercer SW, Norbury M, Watt G, Wyke S et al. (2012) Epidemiology of multimorbidity and implications for health care, research, and medical education: a cross-sectional study. Lancet 380: 37-43. doi:10.1016/S0140-6736(13)60393-1. PubMed: 22579043. 22579043

[2] Boyd CM, Darer J, Boult C, Fried LP, Boult L et al. (2005) Clinical practice guidelines and quality of care for older patients with multiple comorbid diseases: implications for pay for performance. JAMA 294: 716-724. doi:10.1001/jama.294.6.716. PubMed: 16091574. 16091574

[3] Hunt SA, Abraham WT, Chin MH, Feldman AM, Francis GS et al. (2009) 2009 focused update incorporated into the ACC/AHA 2005 Guidelines for the Diagnosis and Management of Heart Failure in Adults: a report of the American College of Cardiology Foundation/American Heart Association Task Force on Practice Guidelines: developed in collaboration with the International Society for Heart and Lung Transplantation. Circulation 119: e391-e479. doi:10.1161/CIRCULATIONAHA.109.192065. PubMed: 19324966. 19324966

[4] Dickstein K, Cohen-Solal A, Filippatos G, McMurray JJ, Ponikowski P et al. (2008) ESC guidelines for the diagnosis and treatment of acute and chronic heart failure 2008: the Task Force for the diagnosis and treatment of acute and chronic heart failure 2008 of the European Society of Cardiology. Developed in collaboration with the Heart Failure Association of the ESC (HFA) and endorsed by the European Society of Intensive Care Medicine (ESICM). Eur J Heart Fail 10: 933-989. doi:10.1016/j.ejheart.2008.08.005. PubMed: 18826876. 18826876

[5] Mann DL, Chakinala M (2011) Heart failure and cor pulmonale. In: DL Longo, AS Fauci, DL Kasper, SL Hauser, JL Jameson et al., editors. Harrison's Principles of Internal Medicine. 18th ed. New York, NY: McGraw-Hill Companies pp. 1901-1915.

[6] McMurray JJ, Pfeffer MA (2012) Heart failure: management and prognosis. In: L Goldman, AI Schafer, editors. Goldman's Cecil Medicine. 24th ed. Philadelphia, PA: Saunders Elsevier pp. 303-318.

[7] Teerlink JR (2008) Diagnosis and management of acute heart failure. In: P Libby, RO Bonow, DL Mann, DP Zipes, editors. Braunwald's Heart Disease: a Textbook of Cardiovascular Medicine. 8th ed. Philadelphia, PA: Saunders Elsevier pp. 583-610.

[8] Abraham WT, Hasan A (2010) Diagnosis and management of heart failure. In: V Fuster, RA Walsh, RA Harrington, editors. Hurst's The Heart. 13th ed. New York, NY: McGraw-Hill Companies pp. 748-780.

[9] Adams KF Jr., Fonarow GC, Emerman CL, LeJemtel TH, Costanzo MR et al. (2005) Characteristics and outcomes of patients hospitalized for heart failure in the United States: rationale, design, and preliminary observations from the first 100,000 cases in the Acute Decompensated Heart Failure National Registry (ADHERE). Am Heart J 149: 209-216. doi:10.1016/j.ahj.2004.08.005. PubMed: 15846257. 15846257

[10] Yancy CW, Lopatin M, Stevenson LW, De Marco T, Fonarow GC (2006) Clinical presentation, management, and in-hospital outcomes of patients admitted with acute decompensated heart failure with preserved systolic function: a report from the Acute Decompensated Heart Failure National Registry (ADHERE) Database. J Am Coll Cardiol 47: 76-84. doi:10.1016/j.jacc.2005.09.022. PubMed: 16386668. 16386668

[11] Fonarow GC, Heywood JT, Heidenreich PA, Lopatin M, Yancy CW (2007) Temporal trends in clinical characteristics, treatments, and outcomes for heart failure hospitalizations, 2002 to 2004: findings from Acute Decompensated Heart Failure National Registry (ADHERE). Am Heart J 153: 1021-1028. doi:10.1016/j.ahj.2007.03.012. PubMed: 17540205. 17540205

[12] O'Connor CM, Starling RC, Hernandez AF, Armstrong PW, Dickstein K et al. (2011) Effect of nesiritide in patients with acute decompensated heart failure. N Engl J Med 365: 32-43. doi:10.1056/NEJMicm1014605. PubMed: 21732835. 21732835

[13] Felker GM, Lee KL, Bull DA, Redfield MM, Stevenson LW et al. (2011) Diuretic strategies in patients with acute decompensated heart failure. N Engl J Med 364: 797-805. doi:10.1056/NEJMoa1005419. PubMed: 21366472. 21366472 PMC3412356

[14] Lichtman JH, Fathi A, Radford MJ, Lin Z, Loeser CS et al. (2006) Acute, severe noncardiac conditions in patients with acute myocardial infarction. Am J Med 119: 843-850. doi:10.1016/j.amjmed.2006.03.040. PubMed: 17000215. 17000215

[15] Barr RG, Bluemke DA, Ahmed FS, Carr JJ, Enright PL et al. (2010) Percent emphysema, airflow obstruction, and impaired left ventricular filling. N Engl J Med 362: 217-227. doi:10.1056/NEJMoa0808836. PubMed: 20089972. 20089972 PMC2887729

[16] Wells JM, Washko GR, Han MK, Abbas N, Nath H et al. (2012) Pulmonary arterial enlargement and acute exacerbations of COPD. N Engl J Med 367: 913-921. doi:10.1056/NEJMoa1203830. PubMed: 22938715. 22938715 PMC3690810

[17] Corrales-Medina VF, Musher DM, Wells GA, Chirinos JA, Chen L et al. (2012) Cardiac complications in patients with community-acquired pneumonia: incidence, timing, risk factors, and association with short-term mortality. Circulation 125: 773-781. doi:10.1161/CIRCULATIONAHA.111.040766. PubMed: 22219349. 22219349

[18] Ray P, Birolleau S, Lefort Y, Becquemin MH, Beigelman C et al. (2006) Acute respiratory failure in the elderly: etiology, emergency diagnosis and prognosis. Crit Care 10: R82. doi:10.1186/cc4926. PubMed: 16723034. 16723034 PMC1550946

[19] Delerme S, Ray P (2008) Acute respiratory failure in the elderly: diagnosis and prognosis. Age Ageing 37: 251-257. doi:10.1093/ageing/afn060. PubMed: 18388161. 18388161

[20] Havranek EP, Masoudi FA, Westfall KA, Wolfe P, Ordin DL et al. (2002) Spectrum of heart failure in older patients: results from the National Heart Failure project. Am Heart J 143: 412-417. doi:10.1067/mhj.2002.120773. PubMed: 11868045. 11868045

[21] Corrales-Medina VF, Suh KN, Rose G, Chirinos JA, Doucette S et al. (2011) Cardiac complications in patients with community-acquired pneumonia: a systematic review and meta-analysis of observational studies. PLOS Med 8: e1001048 PubMed: 21738449. 21738449 10.1371/journal.pmed.1001048 PMC3125176

[22] Hawkins NM, Petrie MC, Jhund PS, Chalmers GW, Dunn FG et al. (2009) Heart failure and chronic obstructive pulmonary disease: diagnostic pitfalls and epidemiology. Eur J Heart Fail 11: 130-139. doi:10.1093/eurjhf/hfn013. PubMed: 19168510. 19168510 PMC2639415

[23] Mentz RJ, Fiuzat M, Wojdyla DM, Chiswell K, Gheorghiade M et al. (2012) Clinical characteristics and outcomes of hospitalized heart failure patients with systolic dysfunction and chronic obstructive pulmonary disease: findings from OPTIMIZE-HF. Eur J Heart Fail 14: 395-403. doi:10.1093/eurjhf/hfs009. PubMed: 22302663. 22302663

[24] Mentz RJ, Schmidt PH, Kwasny MJ, Ambrosy AP, O'Connor CM et al. (2012) The impact of chronic obstructive pulmonary disease in patients hospitalized for worsening heart failure with reduced ejection fraction: an analysis of the EVEREST Trial. J Card Fail 18: 515-523. doi:10.1016/j.cardfail.2012.04.010. PubMed: 22748484. 22748484

[25] Birman-Deych E, Waterman AD, Yan Y, Nilasena DS, Radford MJ et al. (2005) Accuracy of ICD-9-CM codes for identifying cardiovascular and stroke risk factors. Med Care 43: 480-485. doi:10.1097/01.mlr.0000160417.39497.a9. PubMed: 15838413. 15838413

[26] Quan H, Li B, Saunders LD, Parsons GA, Nilsson CI et al. (2008) Assessing validity of ICD-9-CM and ICD-10 administrative data in recording clinical conditions in a unique dually coded database. Health Serv Res 43: 1424-1441. doi:10.1111/j.1475-6773.2007.00822.x. PubMed: 18756617. 18756617 PMC2517283

[27] Kümler T, Gislason GH, Kirk V, Bay M, Nielsen OW et al. (2008) Accuracy of a heart failure diagnosis in administrative registers. Eur J Heart Fail 10: 658-660. doi:10.1016/j.ejheart.2008.05.006. PubMed: 18539522. 18539522

[28] Agency for Healthcare Research and Quality (2006) Statistical brief 2: reasons for being admitted to the hospital through the emergency department, 2003, Healthcare Cost and Utilization Project (HCUP). Available: http://www.hcup-us.ahrq.gov/reports/statbriefs/sb2.pdf. Accessed 25 April 2013.

[29] Lindenauer PK, Pekow PS, Lahti MC, Lee Y, Benjamin EM et al. (2010) Association of corticosteroid dose and route of administration with risk of treatment failure in acute exacerbation of chronic obstructive pulmonary disease. JAMA 303: 2359-2367. doi:10.1001/jama.2010.796. PubMed: 20551406. 20551406

[30] (2007) Expert Panel Report 3 (EPR-3): Guidelines for the diagnosis and management of asthma-summary report 2007. J Allergy Clin Immunol 120: S94-138

[31] Mandell LA, Wunderink RG, Anzueto A, Bartlett JG, Campbell GD et al. (2007) Infectious Diseases Society of America/American Thoracic Society consensus guidelines on the management of community-acquired pneumonia in adults. Clin Infect Dis 44 Suppl 2: S27-S72. doi:10.1086/511159. PubMed: 17278083. 17278083 PMC7107997

[32] Global Initiative for Chronic Obstructive Lung Disease (GOLD) (2011) Global strategy for the diagnosis, management, and prevention of; COPD. Available: http://www.goldcopd.org/uploads/users/files/GOLD_Report_2011_Feb21.pdf. Accessed 25 April 2013. 10.1080/15412555.2017.1394285 29161163

[33] Rothberg MB, Pekow PS, Lahti M, Brody O, Skiest DJ et al. (2010) Antibiotic therapy and treatment failure in patients hospitalized for acute exacerbations of chronic obstructive pulmonary disease. JAMA 303: 2035-2042. doi:10.1001/jama.2010.672. PubMed: 20501925. 20501925

[34] Elixhauser A, Steiner C, Harris DR, Coffey RM (1998) Comorbidity measures for use with administrative data. Med Care 36: 8-27. doi:10.1097/00005650-199801000-00004. PubMed: 9431328. 9431328

[35] Zile MR, Brutsaert DL (2002) New concepts in diastolic dysfunction and diastolic heart failure: Part I: diagnosis, prognosis, and measurements of diastolic function. Circulation 105: 1387-1393. doi:10.1161/hc1102.105289. PubMed: 11901053. 11901053

[36] Uren NG, Davies SW, Jordan SL, Lipkin DP (1993) Inhaled bronchodilators increase maximum oxygen consumption in chronic left ventricular failure. Eur Heart J 14: 744-750. doi:10.1093/eurheartj/14.6.744. PubMed: 8325299. 8325299

[37] Witte KK, Morice A, Cleland JG, Clark AL (2004) The reversibility of increased airways resistance in chronic heart failure measured by impulse oscillometry. J Card Fail 10: 149-154. doi:10.1016/j.cardfail.2003.08.007. PubMed: 15101027. 15101027

[38] Slutsky R (1981) Hemodynamic effects of inhaled terbutaline in congestive heart failure patients without lung disease: beneficial cardiotonic and vasodilator beta-agonist properties evaluated by ventricular catheterization and radionuclide angiography. Am Heart J 101: 556-560. doi:10.1016/0002-8703(81)90221-0. PubMed: 7223595. 7223595

[39] Salpeter SR, Ormiston TM, Salpeter EE (2004) Cardiovascular effects of beta-agonists in patients with asthma and COPD: a meta-analysis. Chest 125: 2309-2321. doi:10.1378/chest.125.6.2309. PubMed: 15189956. 15189956

[40] Au DH, Lemaitre RN, Curtis JR, Smith NL, Psaty BM (2000) The risk of myocardial infarction associated with inhaled beta-adrenoceptor agonists. Am J Respir Crit Care Med 161: 827-830. doi:10.1164/ajrccm.161.3.9904006. PubMed: 10712329. 10712329

[41] Felker GM, O'Connor CM (2001) Inotropic therapy for heart failure: an evidence-based approach. Am Heart J 142: 393-401. doi:10.1067/mhj.2001.117606. PubMed: 11526351. 11526351

[42] Hawkins NM, Petrie MC, Macdonald MR, Jhund PS, Fabbri LM et al. (2011) Heart failure and chronic obstructive pulmonary disease the quandary of Beta-blockers and Beta-agonists. J Am Coll Cardiol 57: 2127-2138. doi:10.1016/j.jacc.2011.02.020. PubMed: 21596228. 21596228

[43] Liu C, Liu G, Zhou C, Ji Z, Zhen Y et al. (2007) Potent diuretic effects of prednisone in heart failure patients with refractory diuretic resistance. Can J Cardiol 23: 865-868. doi:10.1016/S0828-282X(07)70840-1. PubMed: 17876376. 17876376 PMC2651362

[44] Christiansen CF, Christensen S, Mehnert F, Cummings SR, Chapurlat RD et al. (2009) Glucocorticoid use and risk of atrial fibrillation or flutter: a population-based, case-control study. Arch Intern Med 169: 1677-1683. doi:10.1001/archinternmed.2009.297. PubMed: 19822824. 19822824

[45] Wei L, MacDonald TM, Walker BR (2004) Taking glucocorticoids by prescription is associated with subsequent cardiovascular disease. Ann Intern Med 141: 764-770. doi:10.7326/0003-4819-141-10-200411160-00007. PubMed: 15545676. 15545676

[46] Hines LE, Murphy JE (2011) Potentially harmful drug-drug interactions in the elderly: a review. Am J Geriatr Pharmacother 9: 364-377. doi:10.1016/j.amjopharm.2011.10.004. PubMed: 22078863. 22078863

[47] Loo VG, Bourgault AM, Poirier L, Lamothe F, Michaud S et al. (2011) Host and pathogen factors for Clostridium difficile infection and colonization. N Engl J Med 365: 1693-1703. doi:10.1056/NEJMoa1012413. PubMed: 22047560. 22047560

[48] Chen J, Normand SL, Wang Y, Krumholz HM (2011) National and regional trends in heart failure hospitalization and mortality rates for Medicare beneficiaries, 1998-2008. JAMA 306: 1669-1678. doi:10.1001/jama.2011.1474. PubMed: 22009099. 22009099 PMC3688069

[49] Keenan PS, Normand SL, Lin Z, Drye EE, Bhat KR et al. (2008) An administrative claims measure suitable for profiling hospital performance on the basis of 30-day all-cause readmission rates among patients with heart failure. Circ Cardiovasc Qual Outcomes 1: 29-37. doi:10.1161/CIRCOUTCOMES.108.802686. PubMed: 20031785. 20031785

[50] National Quality Forum (2008) National voluntary consensus standards for hospital care 2007: performance measures - a consensus report. Washington, DC: National Quality Forum

[51] Centers for Medicare & Medicaid Services Hospital Pay-for-Performance. Workgroup (2007); US Department of Health and Human Services Medicare Hospital value-based purchasing plan development, issues paper, 1st public listening session. Washington, DC: Centers for Medicare & Medicaid Services.

[52] Rosamond WD, Chang PP, Baggett C, Johnson A, Bertoni AG et al. (2012) Classification of heart failure in the atherosclerosis risk in communities (ARIC) study: a comparison of diagnostic criteria. Circ Heart Fail 5: 152-159. doi:10.1161/CIRCHEARTFAILURE.111.963199. PubMed: 22271752. 22271752 PMC3326579

