# Supplementary material for: Acute Decompensated Heart Failure Is Routinely Treated as a Cardiopulmonary Syndrome
Source: PLoS One. 2013 Oct 21;8(10):e78222. doi: 10.1371/journal.pone.0078222 (PMC3824040; doi:10.1371/journal.pone.0078222)
Supplement: File S1 — Contains Tables S1-S5 and Figure S1. Table S1. Infections Other Than Pneumonia Potentially Requiring Antibiotic Treatment. Table S2. Conditions Other Than Exacerbated Chronic Obstructive Pulmonary Disease Potentially Requiring High-Dose Steroid Treatment. Figure S1. Longitudinal Treatment Pathways. A sample pathway is shown for a patient who receives treatment for heart failure and bronchodilators during the first 2 hospital days and bronchodilators only during hospital days 3 through 5. Within each time period, treatment groups are mutually exclusive and inclusive of all patients. HF, heart failure. Table S3. Treatments Received During the First 2 Hospital Days and Hospital Days 3 through 5. Table S4. Acute Respiratory Treatment During the First Two Days of Hospitalization by Patient Subgroup. Table S5. Longitudinal Treatment Pathways: Treatment During Hospital Days 3 through 5 for Patients within Each Initial Treatment Group. (DOCX) [file pone.0078222.s001.docx]

**Supporting Information for Acute Decompensated Heart Failure is Routinely Treated as a Cardiopulmonary Syndrome**

Kumar Dharmarajan, MD, MBA; Kelly M. Strait, MS; Tara Lagu, MD, MPH; Peter K. Lindenauer, MD, MSc; Mary E. Tinetti, MD; Joanne Lynn, MD; Shu-Xia Li, PhD; Harlan M. Krumholz, MD, SM

**Table S1. Infections Other Than Pneumonia Potentially Requiring Antibiotic Treatment.**

| ICD-9-CM Code | **Description** |
| --- | --- |
| Mycobacterial Disease |  |
| 031.0 | Pulmonary Diseases Due to Other Mycobacteria |
| 031.2 | Disseminated Mycobacterium |
| 031.8 | Other Specified Mycobacterial Diseases |
| 031.9 | Unspecified Diseases Due to Mycobacteria |
| 013.0 | Tuberculous Meningitis |
| 013.3 | Tuberculous Abscess of Brain |
| Meningitis/ Encephalitis |  |
| 036.0 | Meningococcal Meningitis |
| 036.89 | Other Specified Meningococcal Infections |
| 036.9 | Unspecified Meningococcal Infections |
| 320.7 | Meningitis in Other Bacterial Diseases Classified Elsewhere |
| 322.9 | Unspecified Meningitis |
| 320 | Bacterial Meningitis |
| 320.0 | Haemophilus Meningitis |
| 320.1 | Pneumococcal Meningitis |
| 320.2 | Streptococcal Meningitis |
| 320.3 | Staphylococcal Meningitis |
| 320.8 | Meningitis Due to Other Specified Bacteria |
| 320.81 | Anaerobic Meningitis |
| 320.82 | Meningitis Due to Gram-Negative Bacteria, Not Elsewhere Classified |
| 320.9 | Meningitis Due to Unspecified Bacterium |
| 322 | Meningitis of Unspecified Cause |
| V12.42 | Infections of the CNS-Encephalitis, Meningitis |
| Endocarditis |  |
| 421.0 | Acute and Subacute Bacterial Endocarditis |
| 421.9 | Unspecified Acute Endocarditis |
| 424.90 | Endocarditis Valve Unspecified Cause |
| 036.42 | Meningococcal Endocarditis |
| 421.1 | Acute and Subacute Infective Endocarditis in Diseases Classified Elsewhere |
| 422.92 | Septic Myocarditis |
| 424.91 | Endocarditis in Diseases Classified Elsewhere |
| Sinusitis |  |
| 461.0 | Acute Maxillary Sinusitis |
| 461.1 | Acute Frontal Sinusitis |
| 461.2 | Acute Ethmoidal Sinusitis |
| 461.3 | Acute Sphenoidal Sinusitis |
| 461.8 | Other Acute Sinusitis |
| 461.9 | Acute Sinusitis, Unspecified |
| 473.0 | Chronic Maxillary Sinusitis |
| 473.1 | Chronic Frontal Sinusitis |
| 473.2 | Chronic Ethmoidal Sinusitis |
| 473.3 | Chronic Sphenoidal Sinusitis |
| 473.8 | Other Chronic Sinusitis |
| 473.9 | Unspecified Sinusitis |
| Otitis |  |
| 382.0 | Acute Suppurative Otitis Media |
| 382.02 | Acute Suppurative Otitis Media in Diseases Classified Elsewhere |
| 382.3 | Unspecified Chronic Suppurative Otitis Media |
| Oropharyngeal/ Laryngeal Infections |  |
| 462 | Acute Pharyngitis |
| 463 | Acute Tonsillitis |
| 464.0 | Acute Laryngitis |
| 475 | Peritonsillar Abscess |
| 478.21 | Cellulitis of Pharynx or Nasopharynx |
| 478.22 | Parapharyngeal Abscess |
| 478.24 | Retropharyngeal Abscess |
| GI Infections |  |
| 540 | Acute Appendicitis |
| 566 | Abscess of Anal and Rectal Regions |
| 567.2 | Other Suppurative Peritonitis |
| 569.5 | Abscess of Intestine |
| 569.61 | Infection Colostomy or Enterostomy |
| 572.0 | Abscess of Liver |
| 575.0 | Acute Cholecystitis |
| 575.12 | Acute and Chronic Cholecystitis |
| 576.1 | Cholangitis |
| 577.0 | Acute Pancreatitis |
| Urinary Tract Infection |  |
| 599.0 | Urinary Tract Infection Site Not Specified |
| Cellulitis and/or Abscess |  |
| 681.00 | Unspecified Cellulitis and Abscess of Finger |
| 681.10 | Unspecified Cellulitis and Abscess of Toe |
| 682.0 | Cellulitis and Abscess of Face |
| 682.1 | Cellulitis and Abscess of Neck |
| 682.2 | Cellulitis and Abscess of Trunk |
| 682.3 | Cellulitis and Abscess of Upper Arm and Forearm |
| 682.4 | Cellulitis and Abscess of Hand Except Fingers and Thumb |
| 682.5 | Cellulitis and Abscess of Buttock |
| 682.6 | Cellulitis and Abscess Leg Except Foot |
| 682.7 | Cellulitis and Abscess of Foot Except Toes |
| 682.8 | Cellulitis and Abscess of Other Specified Sites |
| 682.9 | Cellulitis and Abscess of Unspecified Sites |
| 681.9 | Cellulitis and Abscess of Unspecified Digit |
| Osteomyelitis |  |
| 730.00 | Acute Osteomyelitis Site Unspecified |
| 730.04 | Acute Osteomyelitis Involving Hand |
| 730.05 | Acute Osteomyelitis Involving Pelvic Region and Thigh |
| 730.07 | Acute Osteomyelitis Involving Ankle and Foot |
| 730.10 | Chronic Osteomyelitis Site Unspecified |
| 730.12 | Chronic Osteomyelitis Involving Upper Arm |
| 730.14 | Chronic Osteomyelitis Involving Hand |
| 730.15 | Chronic Osteomyelitis Involving Pelvic Region and Thigh |
| 730.16 | Chronic Osteomyelitis Involving Lower Leg |
| 730.17 | Chronic Osteomyelitis Involving Ankle and Foot |
| 730.20 | Unspecified Osteomyelitis Site Unspecified |
| 730.24 | Unspecified Osteomyelitis Involving Hand |
| 730.27 | Unspecified Osteomyelitis Involving Ankle and Foot |
| 730.28 | Unspecified Osteomyelitis Involving Other Specified Sites |

ICD-9-CM: International Classification of Diseases, Ninth Revision, Clinical Modification

**Table S2. Conditions Other Than Exacerbated Chronic Obstructive Pulmonary Disease Potentially Requiring High-Dose Steroid Treatment.**

| ICD-9-CM Code | **Description of Condition** |
| --- | --- |
| Skin Disease |  |
| 693 | Dermatitis Due to Substances Taken Internally |
| 693 | Dermatitis Due to Drugs and Medicines Taken Internally |
| 693.1 | Dermatitis Due to Food Taken Internally |
| 693.8 | Dermatitis Due to Other Specified Substances Taken Internally |
| 693.9 | Dermatitis Due to Unspecified Substance Taken Internally |
| 694.4 | Pemphigus |
| 694.5 | Pemphigoid |
| 694.8 | Other Specified Bullous Dermatoses |
| 695 | Erythema Multiforme |
| 695.1 | Erythema Multiforme, Unspecified |
| 695.11 | Erythema Multiforme Minor |
| 695.12 | Erythema Multiforme Major |
| 695.13 | Stevens-Johnson Syndrome |
| 695.14 | Stevens-Johnson Syndrome-Toxic Epidermal Necrolysis Overlap Syndrome |
| 695.15 | Toxic Epidermal Necrolysis |
| 695.19 | Other Erythema Multiforme |
| 695.2 | Erythema Nodosum |
| 695.4 | Lupus Erythematosus |
| 686 | Pyoderma Unspecified |
| 686.01 | Pyoderma Gangrenosum |
| 136.1 | Behcet's Syndrome |
| Collagen Vascular Disease |  |
| 710 | Systemic Lupus Erythematosus |
| 710.1 | Systemic Sclerosis |
| 710.3 | Dermatomyositis |
| 710.4 | Polymyositis |
| 710.8 | Other Specified Diffuse Diseases of Connective Tissue |
| 710.9 | Unspecified Diffuse Connective Tissue Disease |
| 714 | Rheumatoid Arthritis |
| 714.1 | Felty's Syndrome |
| 714.2 | Other Rheumatoid Arthritis With Visceral or Systemic Involvement |
| 725 | Polymyalgia Rheumatica |
| 135 | Sarcoidosis |
| Crystal Arthropathies |  |
| 712 | Crystal Arthropathies |
| 712.1 | Chondrocalcinosis Due to Dicalcium Phosphate Crystals |
| 712.11 | Chondrocalcinosis Due to Dicalcium Phosphate Crystals Involving Shoulder Region |
| 712.12 | Chondrocalcinosis Due to Dicalcium Phosphate Crystals Involving Upper Arm |
| 712.13 | Chondrocalcinosis Due to Dicalcium Phosphate Crystals Involving Forearm |
| 712.14 | Chondrocalcinosis Due to Dicalcium Phosphate Crystals Involving Hand |
| 712.15 | Chondrocalcinosis Due to Dicalcium Phosphate Crystals Involving Pelvic Region And Thigh |
| 712.16 | Chondrocalcinosis Due to Dicalcium Phosphate Crystals Involving Lower Leg |
| 712.17 | Chondrocalcinosis Due to Dicalcium Phosphate Crystals Involving Ankle And Foot |
| 712.18 | Chondrocalcinosis Due to Dicalcium Phosphate Crystals Involving Other Specified Sites |
| 712.19 | Chondrocalcinosis Due to Dicalcium Phosphate Crystals Involving Multiple Sites |
| 712.2 | Chondrocalcinosis Due to Pyrophosphate Crystals |
| 712.2 | Chondrocalcinosis Due to Pyrophosphate Crystals Involving Unspecified Site |
| 712.21 | Chondrocalcinosis Due to Pyrophosphate Crystals Involving Shoulder Region |
| 712.22 | Chondrocalcinosis Due to Pyrophosphate Crystals Involving Upper Arm |
| 712.23 | Chondrocalcinosis Due to Pyrophosphate Crystals Involving Forearm |
| 712.24 | Chondrocalcinosis Due to Pyrophosphate Crystals Involving Hand |
| 712.25 | Chondrocalcinosis Due to Pyrophosphate Crystals Involving Pelvic Region And Thigh |
| 712.26 | Chondrocalcinosis Due to Pyrophosphate Crystals Involving Lower Leg |
| 712.27 | Chondrocalcinosis Due to Pyrophosphate Crystals Involving Ankle And Foot |
| 712.28 | Chondrocalcinosis Due to Pyrophosphate Crystals Involving Other Specified Sites |
| 712.29 | Chondrocalcinosis Due to Pyrophosphate Crystals Involving Multiple Sites |
| 712.3 | Chondrocalcinosis Cause Unspecified |
| 712.3 | Chondrocalcinosis Cause Unspecified Involving Unspecified Site |
| 712.31 | Chondrocalcinosis Cause Unspecified Involving Shoulder Region |
| 712.32 | Chondrocalcinosis Cause Unspecified Involving Upper Arm |
| 712.33 | Chondrocalcinosis Cause Unspecified Involving Forearm |
| 712.34 | Chondrocalcinosis Cause Unspecified Involving Hand |
| 712.35 | Chondrocalcinosis Cause Unspecified Involving Pelvic Region And Thigh |
| 712.36 | Chondrocalcinosis Cause Unspecified Involving Lower Leg |
| 712.37 | Chondrocalcinosis Cause Unspecified Involving Ankle And Foot |
| 712.38 | Chondrocalcinosis Cause Unspecified Involving Other Specified Sites |
| 712.39 | Chondrocalcinosis Cause Unspecified Involving Multiple Sites |
| 712.8 | Other Specified Crystal Arthropathies |
| 712.8 | Other Specified Crystal Arthropathies Site Unspecified |
| 712.81 | Other Specified Crystal Arthropathies Involving Shoulder Region |
| 712.82 | Other Specified Crystal Arthropathies Involving Upper Arm |
| 712.83 | Other Specified Crystal Arthropathies Involving Forearm |
| 712.84 | Other Specified Crystal Arthropathies Involving Hand |
| 712.85 | Other Specified Crystal Arthropathies Involving Pelvic Region And Thigh |
| 712.86 | Other Specified Crystal Arthropathies Involving Lower Leg |
| 712.87 | Other Specified Crystal Arthropathies Involving Ankle And Foot |
| 712.88 | Other Specified Crystal Arthropathies Involving Other Specified Sites |
| 712.89 | Other Specified Crystal Arthropathies Involving Multiple Sites |
| 712.9 | Unspecified Crystal Arthropathy |
| 712.9 | Unspecified Crystal Arthropathy Site Unspecified |
| 712.91 | Unspecified Crystal Arthropathy Involving Shoulder Region |
| 712.92 | Unspecified Crystal Arthropathy Involving Upper Arm |
| 712.93 | Unspecified Crystal Arthropathy Involving Forearm |
| 712.94 | Unspecified Crystal Arthropathy Involving Hand |
| 712.95 | Unspecified Crystal Arthropathy Involving Pelvic Region And Thigh |
| 712.96 | Unspecified Crystal Arthropathy Involving Lower Leg |
| 712.97 | Unspecified Crystal Arthropathy Involving Ankle And Foot |
| 712.98 | Unspecified Crystal Arthropathy Involving Other Specified Sites |
| 712.99 | Unspecified Crystal Arthropathy Involving Multiple Sites |
| Vasculitis |  |
| 446 | Polyarteritis Nodosa |
| 446.1 | Acute Febrile Mucocutaneous Lymph Node Syndrome (MCLS) |
| 446.2 | Hypersensitivity Angiitis |
| 446.2 | Hypersensitivity Angiitis Unspecified |
| 446.21 | Goodpasture's Syndrome |
| 446.29 | Other Specified Hypersensitivity Angiitis |
| 446.4 | Wegener's Granulomatosis |
| 446.5 | Thrombotic Microangiopathy |
| 446.7 | Takayasu's Disease |
| 443.1 | Thromboangiitis Obliterans (Buerger's Disease) |
| Gastrointestinal Disease |  |
| 556 | Ulcerative Enterocolitis |
| 556 | Ulcerative (Chronic) Enterocolitis |
| 556.1 | Ulcerative (Chronic) Ileocolitis |
| 556.2 | Ulcerative (Chronic) Proctitis |
| 556.3 | Ulcerative (Chronic) Proctosigmoiditis |
| 556.4 | Pseudopolyposis of Colon |
| 556.5 | Left‑Sided Ulcerative (Chronic) Colitis |
| 556.6 | Universal Ulcerative (Chronic) Colitis |
| 556.8 | Other Ulcerative Colitis |
| 556.9 | Ulcerative Colitis Unspecified |
| 555 | Regional Enteritis |
| 555 | Regional Enteritis of Small Intestine |
| 555.1 | Regional Enteritis of Large Intestine |
| 555.2 | Regional Enteritis of Small Intestine with Large Intestine |
| 555.9 | Regional Enteritis of Unspecified Site |
| 571.42 | Autoimmune Hepatitis |
| Hematologic Disease |  |
| 203 | Multiple Myeloma |
| 203 | Multiple Myeloma without Remission |
| 203.01 | Multiple Myeloma in Remission |
| 203.02 | Multiple Myeloma, in Relapse |
| 287.31 | Immune Thrombocytopenic Purpura |
| 283 | Autoimmune Hemolytic Anemias |
| 284.89 | Red Cell Aplasia (Acquired) (Adult) (with Thymoma) |
| 284.9 | Aplastic Anemia Unspecified |
| Neurologic Disease |  |
| 357.81 | Chronic Inflammatory Demyelinating Polyneuritis |
| 357 | Acute Infective Polyneuritis |
| 341.2 | Acute (Transverse) Myelitis |
| 341.2 | Acute (Transverse) Myelitis Not Otherwise Specified |
| 341.21 | Acute (Transverse) Myelitis in Conditions Classified Elsewhere |
| 341.22 | Idiopathic Transverse Myelitis |
| 340 | Multiple Sclerosis |
| Endocrine Disease |  |
| 255.4 | Corticoadrenal Insufficiency |
| 255.41 | Glucocorticoid Deficiency |
| Renal Disease |  |
| 581.3 | Nephrotic Syndrome with Lesion of Minimal Change Glomerulonephritis |
| 583.9 | Nephritis and Nephropathy Not Specified as Acute or Chronic With Unspecified Pathological Lesion in Kidney |
| Cardiac Disease |  |
| 420 | Acute Pericarditis |
| 420 | Acute Pericarditis in Diseases Classified Elsewhere |
| 420.9 | Other and Unspecified Acute Pericarditis |
| 420.91 | Acute Idiopathic Pericarditis |
| 420.99 | Other Acute Pericarditis |
| 422 | Acute Myocarditis |
| 422 | Acute Myocarditis in Diseases Classified Elsewhere |
| 422.9 | Other and Unspecified Acute Myocarditis |
| 422.9 | Acute Myocarditis Unspecified |
| 422.91 | Idiopathic Myocarditis |
| Transplant-related Conditions |  |
| 996.8 | Complications f Transplanted Organ |
| 996.8 | Complications of Unspecified Transplanted Organ |
| 996.81 | Complications of Transplanted Kidney |
| 996.82 | Complications of Transplanted Liver |
| 996.83 | Complications of Transplanted Heart |
| 996.84 | Complications of Transplanted Lung |
| 996.85 | Complications of Transplanted Bone Marrow |
| 996.86 | Complications of Transplanted Pancreas |
| 996.87 | Complications of Transplanted Organ Intestine |
| 996.89 | Complications of Other Specified Transplanted Organ |
| Allergy-related Conditions |  |
| 995 | Other Anaphylactic Reaction Not Elsewhere Classified |
| 995.1 | Angioneurotic Edema Not Elsewhere Classified |
| 995.2 | Unspecified Adverse Effect of Drug Medicinal and Biological Substance Not Elsewhere Classified |
| 995.2 | Unspecified Adverse Effect of Unspecified Drug, Medicinal and Biological Substance |
| 995.27 | Other Drug Allergy |
| 995.3 | Allergy Unspecified Not Elsewhere Classified |

ICD-9-CM: International Classification of Diseases, Ninth Revision, Clinical Modification

**Figure S1.**

**Treatment During Days 1-2 Treatment During Days 3-5**

**
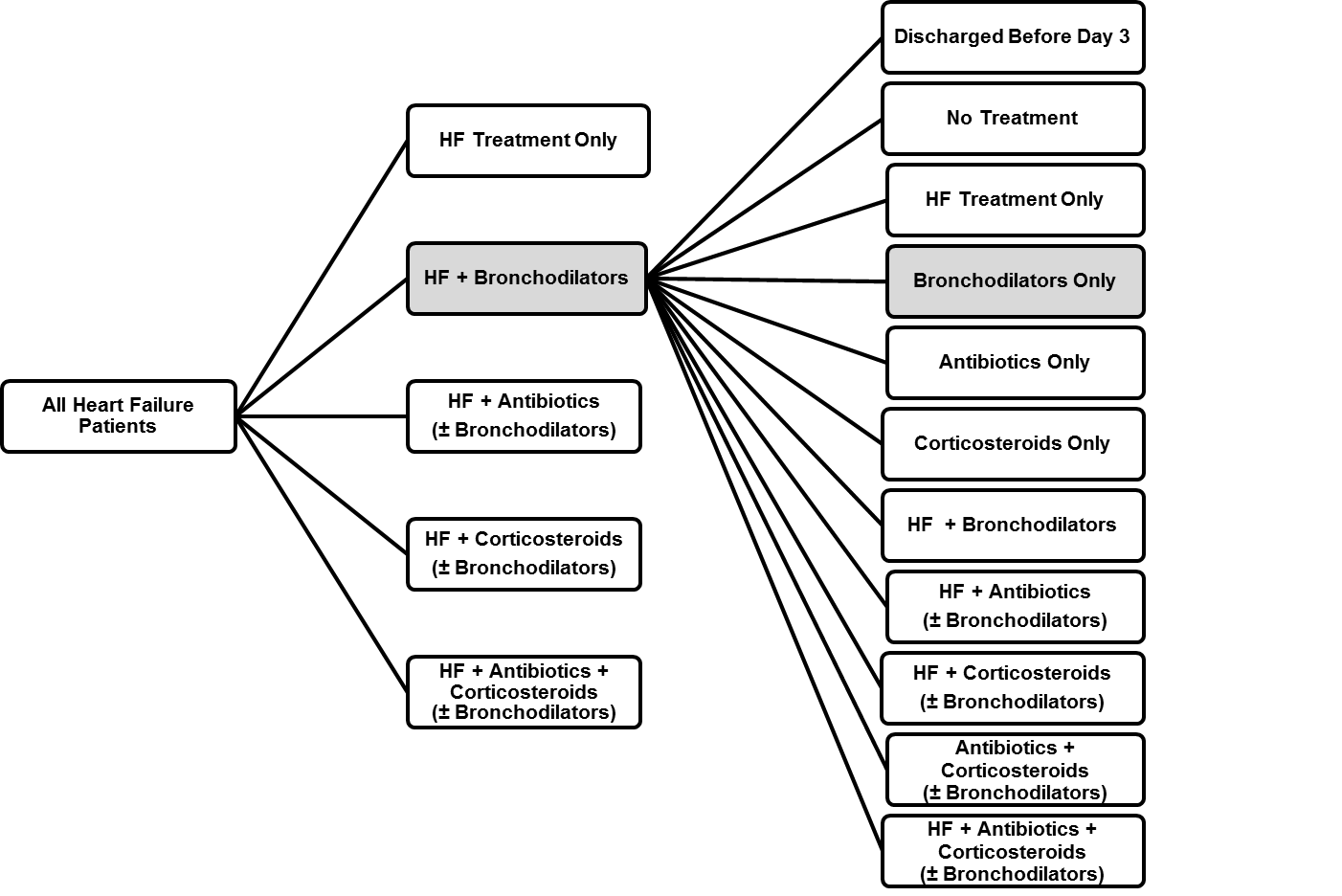
**

Longitudinal Treatment Pathways. A sample pathway is shown for a patient who receives treatment for heart failure and bronchodilators during the first 2 hospital days and bronchodilators only during hospital days 3 through 5. Within each time period, treatment groups are mutually exclusive and inclusive of all patients.

HF, heart failure.

**Table S3. Treatments Received During the First 2 Hospital Days and Hospital Days 3 through 5.**

| Treatment Category | | **All Heart Failure Hospitalizations (N=164,494)** | | **Heart Failure Hospitalizations with Chronic Lung Disease (N=63,690)** | |
| --- | --- | --- | --- | --- | --- |
|  | | **Percent of Patients Receiving Treatment During Days 1-2**  **(N=164,494)** | **Percent of Patients Receiving Treatment During Days 3-5**  **(N=157,830)** | **Percent of Patients Receiving Treatment During Days 1-2**  **(N=63,690)** | **Percent of Patients Receiving Treatment During Days 3-5**  **(N=61,948)** |
| Heart Failure Treatments | **Oral Loop Diuretics** | 28% | 52% | 29% | 52% |
|  | **Intravenous Loop Diuretics** | 90% | 63% | 90% | 65% |
|  | **Intravenous Inotropes** | 5% | 6% | 5% | 5% |
|  | **Intravenous Vasodilators** | 7% | 2% | 7% | 2% |
| Short-acting Inhaled Bronchodilators | **Beta Agonists** | 35% | 28% | 56% | 47% |
|  | **Anti-Cholinergics** | 28% | 21% | 45% | 36% |
|  | **Other** | 1% | 1% | 2% | 2% |
|  | **Any Bronchodilator** | 37% | 30% | 59% | 50% |
| Antibiotics | **Penicillins** | 4% | 4% | 5% | 5% |
|  | **Cephalosporins** | 13% | 11% | 15% | 13% |
|  | **Fluoroquinolones** | 16% | 15% | 22% | 20% |
|  | **Macrolides** | 9% | 7% | 12% | 10% |
|  | **Tetracyclines** | 1% | 1% | 1% | 2% |
|  | **Vancomycin** | 4% | 4% | 5% | 5% |
|  | **Aminoglycosides** | 0% | 0% | 0% | 0% |
|  | **Carbapenems** | 0% | 1% | 3% | 1% |
|  | **Other** | 2% | 3% | 11% | 3% |
|  | **Any Antibiotic** | 33% | 33% | 42% | 42% |
| High-dose Corticosteroids | **Intravenous Corticosteroids** | 6% | 1% | 11% | 2% |
|  | **Oral Corticosteroids** | 3% | 3% | 6% | 5% |
|  | **Any Corticosteroid** | 10% | 5% | 17% | 9% |

**Table S4: Acute Respiratory Treatment During the First Two Days of Hospitalization by Patient Subgroup.**

| Patient Subgroup | | **Number of Hospitalizations** | **Initial Treatment (Hospital Days 1-2)** | | | | |
| --- | --- | --- | --- | --- | --- | --- | --- |
|  |  |  | **HF Only** | **HF + Bronchodilators** | **HF + Antibiotics (± Bronchodilators)** | **HF + Corticosteroids (± Bronchodilators)** | **HF + Antibiotics + Corticosteroids (± Bronchodilators)** |
| Age in Years | **<50** | 11,522 | 53% | 16% | 24% | 3% | 4% |
|  | **50-70** | 50,893 | 47% | 18% | 25% | 4% | 6% |
|  | **>70** | 102,079 | 47% | 16% | 29% | 3% | 5% |
| History of Acute Myocardial Infarction | **Yes** | 5,464 | 40% | 16% | 34% | 3% | 6% |
|  | **No** | 159,030 | 47% | 17% | 27% | 4% | 5% |
| History of Chronic Lung Disease | **Yes** | 63,690 | 27% | 25% | 31% | 6% | 11% |
|  | **No** | 100,804 | 59% | 12% | 25% | 2% | 2% |
| Daily Treatment with Loop Diuretics During Days 3-5 | **Yes** | 143,576 | 47% | 17% | 27% | 4% | 5% |
|  | **No** | 20,918 | 48% | 15% | 28% | 4% | 5% |
| Natriuretic Peptide Testing During Days 1-2 | **Yes** | 142,123 | 46% | 17% | 27% | 4% | 6% |
|  | **No** | 22,371 | 50% | 15% | 28% | 3% | 4% |
| Chest Radiograph During Days 1-2 | **Yes** | 149,573 | 46% | 17% | 28% | 4% | 5% |
|  | **No** | 14,921 | 57% | 15% | 22% | 3% | 3% |

HF, heart failure

**Table S5. Longitudinal Treatment Pathways: Treatment During Hospital Days 3 through 5 for Patients within Each Initial Treatment Group.**

| **Continuing Treatment (Hospital Days 3-5);**  **(% of patients)** | **Initial Treatment (Hospital Days 1-2)**  **(N=164,494)** | | | | |
| --- | --- | --- | --- | --- | --- |
|  | **HF Only; (n=77,175)** | **HF + Bronchodilators; (n=27,980)** | **HF + Antibiotics**  **(± Bronchodilators); (n=44,846)** | **HF + Corticosteroids**  **(± Bronchodilators); (n=5,852)** | **HF + Antibiotics + Corticosteroids**  **(± Bronchodilators); (n=8,641)** |
| **Discharged Before Day 3** | **6%** | 3% | 2% | 3% | 1% |
| **No Continuing Treatment** | **25%** | 13% | 11% | 15% | 7% |
| **HF only** | **69%** | 35% | 20% | 29% | 11% |
| **Bronchodilators Only** | **0%** | 12% | 4% | 9% | 6% |
| **Antibiotics Only**  **(± Bronchodilators)** | **0%** | 0% | 15% | 0% | 12% |
| **Corticosteroids Only**  **(± Bronchodilators)** | **0%*** | 0%* | 0%* | 6% | 3% |
| **HF + Bronchodilators** | **0%** | 35% | 7% | 19% | 10% |
| **HF + Antibiotics**  **(± Bronchodilators)** | **0%** | 0% | 37% | 0% | 25% |
| **HF + Corticosteroids**  **(± Bronchodilators)** | **0%*** | 1% | 1% | 19% | 7% |
| **Antibiotics + Corticosteroids**  **(± Bronchodilators)** | **0%** | 0% | 0%* | 0% | 5% |
| **HF + Antibiotics + Corticosteroids**  **(± Bronchodilators)** | **0%** | 0% | 1% | 0% | 14% |

*Patients in these categories comprised less than 1% of the total population.

Cell percentages reflect the division of patients within each of the 5 initial treatment groups into 1 of 11 continuing treatment pathways. Pathways are mutually exclusive and exhaustive of all patients within the study sample. Darker cells have a larger percentage of patients.

HF: heart failure
